# Investigation of spiral-wound membrane modules for the cross-flow nanofiltration of fermentation broth obtained from a pilot plant fermentation reactor for the continuous production of lactic acid

**DOI:** 10.1186/s40643-016-0133-5

**Published:** 2017-01-04

**Authors:** Hendrik Laube, Roland Schneider, Joachim Venus

**Affiliations:** Department of Bioengineering, Leibniz-Institute for Agricultural Engineering Potsdam-Bornim e.V., Max-Eyth-Allee 100, 14469 Potsdam, Brandenburg Germany

**Keywords:** Nanofiltration, Sodium lactate, Spiral-wound modules, Cross-flow, Pilot plant

## Abstract

**Background:**

The separation performance of seven polymer membranes for the nanofiltration of sodium lactate in fermentation broth was investigated. Each module was introduced into the test stand, and the system curve was obtained by recording the permeate flow velocity at different pump head levels. Performance benchmarks were good permeate quality, as determined by high permeate flow velocity, high sodium lactic concentration, low ion impurity concentration, and low organic impurity concentration. Market research has shown that three companies, DOW (TW30, SW30, NF45), General Electric (DK73, DL73), and Microdyn-Nadir (NP30), distributed spiral-wound membrane modules for cross-flow filtration in a 2.5 by 40-in. module size, suitable for operation in the filtration test stand.

**Results:**

The measured permeate flow velocity was found to vary widely between the membranes. At a pump head of 250 m, DK73, NP30, and DL73 generated more than 200, 300, and 400% higher permeate flow velocities, respectively, than TW30 and NF45. A key benchmark, lactate rejection, was also highly dependent upon membrane type. The NP30, NF45, and TW30 membranes showed a decrease in lactate permeate flow velocity of 117, 83, and 348% starting at 205, 250, and 300 m, respectively.

**Conclusions:**

The DL73 had the overall best performance according to the measured fermentation broth and lactic acid permeability. The presented method for the graphical analysis of the membrane performance proofed to be a useful tool for the filtration engineer.

**Electronic supplementary material:**

The online version of this article (doi:10.1186/s40643-016-0133-5) contains supplementary material, which is available to authorized users.

## Background

With the ongoing scarcity of fossil-based fuels and chemicals, mankind has been driven to find new substitutes for petrochemical-based polymers (Eerkens 2010). Lactic acid (LA) has been identified by the Department of Energy (USA) as one of the top five bio-based monomers for the production of bio-polymers (Werpy et al. 2007). The first large-scale production of LA by bioprocessing was first reported in 2005 (Vink et al. 2010). Cross-flow membrane filtration is now a mature technology (Kamm et al. 2008). Applications include liquid processing to effect clarification, product isolation, concentration and/or separation (Gautam and Menkhaus 2014). Selective, consistent separation, and the increased product yield in combination with a low energy consumption have made cross-flow filtration a suitable technique for the downstream processing of high-volume chemicals obtained from petrochemicals and fermentation processes (Abels et al. 2013). In conventional filtration, the feed flow is perpendicular to the membrane surface (Mänttäri et al. 2013). This causes a buildup of debris, which eventually reduces fluid permeation. When the flow of the feed stream is tangential to the membrane surface, as it is in cross-flow filtration, the kinetic force of the stream results in a continuous scouring action, which is strong enough to eliminate almost any formation of a membrane fouling layer produced by debris and macromolecules contained in the feed stream (Carrère et al. 2001).

Pressure is the driving force of the filtration process (Pabby et al. 2009). The fraction of the feed stream that passes through the membrane is called the “permeate” or “filtrate.” The fraction that does not pass through the membrane is called the “retentate” or the “concentrate” (Crespo and Böddeker 1994). In the case of sodium lactate filtration for the production of LA, the feed stream is the fermentation broth from either a batch or a continuous fermentation process (Carrère et al. 2001). It has a high sodium lactate (61,800 ppm) and a medium macromolecule concentration. The permeate has a medium sodium lactate (57,000 ppm) and a low macromolecule concentration. The retentate has a low sodium lactate (35,300 ppm) and a high macromolecule concentration (Laube et al. 2016a). The retentate, in the case of the continuous process, is either recycled back to the fermenter, along with the LA bacteria, or forwarded to another membrane separator (Kamm et al. 2008). This procedure is repeated until a low lactate concentration of around 35,300 ppm is reached in the retentate stream. The permeate is further processed and not recycled to the fermenter. As a consequence, the macromolecules contained in the fermenter are continuously concentrated (Sikder et al. 2012). There is a wide range of cross-flow filtration modules available on the market, and the appropriate module depends on the size and type of the macromolecules, particles, and solutes contained in the feed (Porter 1990). Laboratory experiments, with just the plane membrane film, can yield only a little information on the performance of the later constructed membrane module, such as the basic yield of a given molecule, making pilot plant experiments under production conditions mandatory (Koschuh et al. 2005). For separation technologists to choose the right membrane, they have to explore individually how each membrane best performs the clarification task in the given process (Gautam and Menkhaus 2014).

In recent years, the simulation of membrane modules performances has come into the focus of the scientist community. A rising number of experiments can enhance the information on the membrane, to a certain point. However, in order to adequately simulate a membrane performance, a broad set of experiments and instruments are necessary. For a research engineer in the field of bio-processing and bio-separation, who has make an economical, rational decision for the benefit of his overall production process, the parameters for membrane simulation are time consuming and not of greater value. As the primary cost drivers are the product yield (high product concentration in the permeate stream) and a low pump head (operational, electrical cost of the pump). These two parameters can be obtained using a regular filtration unit and our hereby presented mathematical and graphical procedure. The applied feed streams were deionized water and microfiltered fermentation broth, containing the desired product, sodium lactate.

Within this study, seven spiral-wound, cross-flow filtration modules were investigated for their performance on the filtration of sodium lactate from fermentation broth. The membrane performances were evaluated in terms of permeate flow velocity, high lactate permeability, and low macromolecule permeability. The target was to determine the most efficient membrane with a reasonable number of experiments. The presented method is applicable beyond this particular bio-processing system, for other filtration systems, where the product is desired to be located in the permeate stream, and especially for small molecular weight organic acids.

## Methods

### Instrumentation and analytical equipment

Lactate concentration was analyzed by HPLC (Dionex Ultimate 3000) with the column Eurokat H (Knauer 300 × 8 mm, 10 µm). The mobile phase consisted of 0.01 N H_2_SO_4_, with a flow of 0.8 ml min^−1^ and a pressure of 65 bar, with a temperature of 40 °C. A refractive index detector (Shodex RI-101) was used with an auto sampler (Analytical WPS-3000TSL) and an injection volume of 10 µl.

Inorganic anion concentration was determined by ion chromatography (Dionex ICS 1000) with the suppressed conductivity ASRS-Ultra detector. For the anion measurement, the IonPac AG9-HC column (pre column 4 × 50 mm) and IonPac AS9-HC (separation column 4 × 250 mm) were used with the mobile phase consisting of 9 mmol Na_2_CO_3_ and a flow of 1.2 ml min^−1^. The pressure was set to 160 bar at room temperature. The injection volume of 25 µl and the AS40 (Dionex) auto sampler were applied.

Inorganic cation concentration was analyzed by ion chromatography (Dionex ICS 1000) with the suppressed conductivity ASRS-Ultra detector. For the cation measurement, the IonPac CG 16 column (pre column 5 × 50 mm) and IonPac CS 16 column (separation column 5 × 250 mm) were used. The mobile phase consisted of 30 mmol MSA and the permeate flux was set to 1.0 ml min^−1^. The pressure was set to 80 bar with a temperature of 40 °C.

Organic fractions in the complex sample matrix were measured by capillary electrophoresis (CE) (Agilent 7100 Capillary Electrophoresis System) with UV detection. The electrolyte consisted of two additives, 25 mM borate, and 50 mM sodium dodecyl sulfate (SDS). The capillary employed was a bare fused silica bubble cell with an inner diameter of 50 µm and length of 64 cm. The injection was carried out under the following conditions. The injection time was set to 10 s. The pressure was set to 50 mbar. The temperature was set to 25 °C. The current was set to −30 kV. The direct detection mode micellar electrokinetic chromatography (MEKC) was applied. The detection wavelength and bandwidth were each 200 nm (Laube et al. 2016).

### Experimental apparatus and procedure

The filtration plant, shown in Additional file 1: Figure S1, was constructed by the UFI-TEC company, Germany. At the beginning of each experiment, deionized water was used to measure the water permeate flux. At each pressure level, the permeate flux was measured four times and the arithmetic average was calculated. The feed pump installed into the plant provided a maximum pump head of 500 m. The pump head was raised by 50-m steps, starting from an initial membrane pump head of 100 m, until the individual maximum flux level of each membrane was reached. Each pump head level was maintained for 30 min; then, a sample from the permeate was taken. The permeate was collected in a 5-l measuring cup and forwarded into a 60-l plastic tank. Then, an additional 5 l of feed was provided to the feed tank. The permeate flux velocity was measured every two min by using a 250-ml graduated cylinder and a stopwatch for 10 or 30 s. After the experiment, a sample from the retentate was taken. The maximum pressures for the individual membranes are listed in Table 1. The temperature was set to 25 °C. The feed tank was filled with 25 l of microfiltered fermentation broth. The feed was pumped through a preliminary Teflon fabric filter and then fed to the membrane module. The two-way valve on the feed line was adjusted such that the flow meter for the membrane module indicated a cross-flow velocity of 300 l h^−1^ at 20 °C. The rejection was calculated according to Eq. (1) in Additional file 1: Table S2.

**Table 1:** Membrane characteristics taken from the producers’ factsheets, testing conditions of the membrane according to the fact sheets (n.s.) for not specified and the calculated Reynolds numbers according to the flow-through velocity for each membrane

Membrane	DK73	DL73	NP30	NF45	NF90	TW30	SW30
NMWCO (g/mol)	150–300		ns	200	ns	ns	ns
Minimum pH (–)	ns	ns	0	3	2	2	2
Maximum pH (–)	ns	ns	14	10	11	11	11
Maximum temperature (°C)	50	50	95	45	40	45	45
Maximum pressure (bar)	41.4	41.5	40	41	41	41	69
Active area (m^2^)	1.6	1.7	1.8	2.6	2.6	2.6	2.8
Feed spacer thickness (mm)	1.27	1.27	1.1	ns	ns	0.7	ns
Permeate flow rate (m^3^/d)	1.3	1.7	1.728	3.5	2.3	3.2	2.6
Salt rejection (%)	98	96	80–95	98	90	99.5	99.4
Substance (–)	MgSO_4_	MgSO_4_	Na_2_SO_4_	MgSO_4_	MgSO_4_	NaCl	NaCl
Concentration (ppm)	2.000	2.000	ns	2.000	2.000	2.000	32.000
Temperature (°C)	25	25	20	25	25	25	25
Duration (h)	24	24	ns	ns	ns	ns	ns
Variation (± X %)	25	25	ns	ns	ns	ns	ns
Recovery (%)	15	15	ns	15	15	15	8
Pressure (bar)	7.6	7.6	40	8.9	5	15.5	55
Permeate flux (m^3^/d)	1.3	1.7	1.728	3.5	2.3	3.2	2.6
Membrane width (m)	1.57	1.67	1.77	2.56	2.56	2.56	2.76
Feed spacer thickness (mm)	1.27	1.27	1.1	0.9	0.9	0.7	0.9
(m)	0.001	0.001	0.001	0.001	0.001	0.001	0.001
Feed spacer area (m^2^)	0.002	0.002	0.002	0.002	0.002	0.002	0.002
Circular area diameter (m)	0.050	0.052	0.050	0.054	0.054	0.048	0.056
Flow velocity (m/s)	0.042	0.039	0.043	0.036	0.036	0.047	0.034
Reynolds number (–)	2624	2546	2659	2445	2445	2773	2357
Friction losses (–)	0.046	0.047	0.046	0.047	0.047	0.045	0.048
B (–)	0.939	0.920	0.9472	0.896	0.896	0.974	0.874

Membranes with a low pressure loss have a flat curve and hence have a high permeate flow velocity at moderate pump head values. This is the desired performance, as it allows for the generation of great quantities of filtered permeate at low operational costs. The pressure built up on the feed side is generated by the hydraulic performance of the pump, which again is proportional to the axial rotation of the impeller, which requires the force of the electrical motor.

### Market investigation, membrane selection, and properties

Market investigation for suitable membrane modules was carried out with the following criteria: The membrane had to be commercially available in Germany. The membranes had to fit into cartridges with dimension of 2.5 in. (6.35 cm) in diameter and 40 in. (101.6 cm) in length. This was necessary for installation into the pilot plant. Nanofiltration membranes for the explicit application of the filtration of small molecular compounds (e.g., organic acids) in the permeate were preferred. The following membranes were acquired: from General Electric (GE Power & Water), model DK73 (DK2540F1073) and DL73 (DL2540F1073); from Microdyn-Nadir, model NP30 (Nadir NP030P); from Dow Chemical Company (Dow Water & Process Solutions), model TW30 (FILMTEC TW30-2549), SW30 (FILMTEC SW 30-2540), and NF45 (FILMTEC NF45-2540). All membranes were made of polyamide thin film composite material. The membranes’ characteristics, obtained from the producer’s fact sheets, and the testing conditions are listed in Table 1.

The DL series membrane was designed for industrial high flow nanofiltration with a minimum rejection of 96%, and the DK series membrane for industrial high rejection nanofiltration with a minimum rejection of 98%. Both were designed for dye removal and concentration; sodium chloride diafiltration; and metals recovery (Electric 2014). The NP30 membrane was designed for the preparation of acid and caustic solutions (Microdyn-Nadir 2014). The NF45 membrane was labeled as a desalting nanofiltration element for process streams, including applications such as the desalting of organic compounds, acid processing, metal recovery, and antifreeze recovery (Dow 2014a). The NF90 membrane was designed for the removal of high salt and iron concentrations and normal pesticide, herbicide, and total organic carbon (TOC) levels (Dow 2014b). The TW30 membrane was designed for the production of the highest quality water, and the SW30 membrane was designed for seawater reverse osmosis, specifically to obtain the highest flow rates for sea and land-based desalination (Dow 2014c).

### Mathematic and graphic analysis

**Fig. 1:**
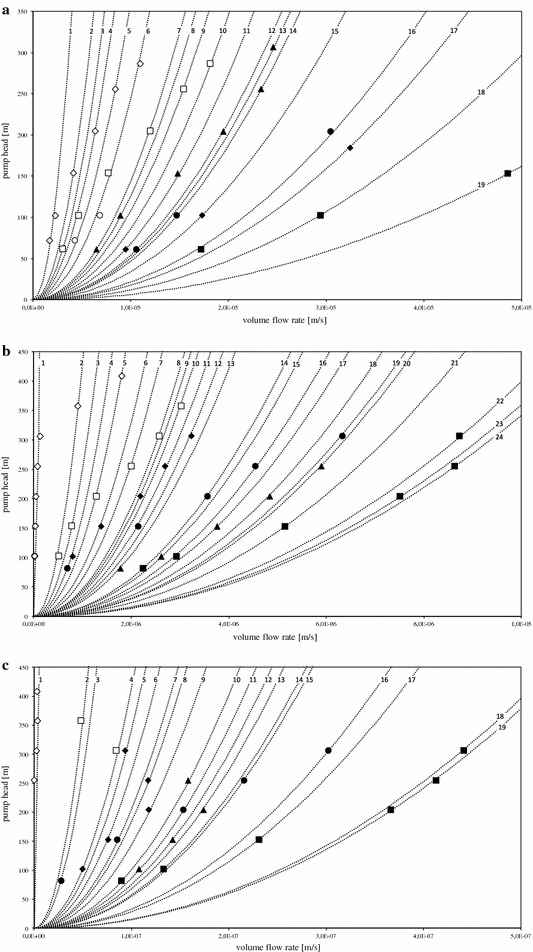
The measured permeate flow velocity for each membrane at different levels of applied pump head. **a** Permeate flow velocity of deionized water, **b** of media, and **c** of the LA contained in the media. The system curves are represented as* dotted lines*, the membranes are represented as follows: *black fill*: DL73, *square*; NF45, *rhombus*; DK73, *circle*; NP30, *triangle*; *white fill*: TW30, *square*; SW30, *rhombus*; NF90, *circle*. The figure allows for the comparison of the optimal operation window amongst the membranes. A high permeate flow velocity (*far right*) at a low pump head (near the * x-axis*) are desired. For example, in **a** the membrane DL73 has a three-fold better permeate flow velocity at a pump head of 100 m than the membrane NP30

Each module was introduced into the test stand and the system curve was obtained by recording the permeate volume flow ($\dot{V}$) in [ml s^−1^] at different feed pressure levels. The permeate flow was divided by the individual membrane area (*A*) in [m^2^] to yield the permeate flow velocity (*ϑ*) in [m s^−1^]. The set feed pressure level (*P*) in [bar] was converted into the pump head losses (*H*_*Jt*_) in [*m*], using the water density (*ρ*) in [kg m^−3^] at the experimental conditions of 30 °C and the gravity constant (g) in [m s^−2^]. The measured values of each membrane were plotted with the pump head losses as a function of the permeate flow velocity. The plots are given in Fig. 1a with deionized water as feed, in Fig. 1b with fermentation broth as feed, and in Fig. 1c with the sodium lactate contained in the fermentation broth permeate stream. The following equations can be found in the common literature on the friction losses of pumps as well as in Additional file 1: Table S2 (Karassik 2008; Aktiengesellschaft 2010). Both parameters stand in a quadratic relation by Eq. (1) in Additional file 1: Table S2. Equation (1) has the cubic form of Eq. (2) in Additional file 1: Table S2.

The dimensionless friction losses (*λ*) in [–] are calculated by Eq. (3) in Additional file 1: Table S2 (Rothbart and Brown 2006). The Reynolds number (*Re*_*d*_) in [–] is calculated by Eq. (4) in Additional file 1: Table S2, using the internal flow velocity (*ϑ*_*i*_) in [m s^−1^] within the membrane module; the characteristic diameter (*d*_*i*_) in [*m*] defined as the horizontal inlet area generated by the feed spacer height and the membrane depth (see Table 1); and the dynamic viscosity (*υ*) in [kg m^−1^s^−1^], or the kinematic viscosity (*η*) in [m^2^ s^−1^] of water at 30 °C (Reynolds 1883; Mays 1999). With the cubic form of (2), *a* equals as in Eq. (5) in Additional file 1: Table S2.

The parameter *a* can also be obtained through the gradient of the system curve, which intersects the measured values of the membranes at exactly one point. Hence, the dimensionless summary score of all pressure losses for shaped pieces and fittings in the system (∑ *ξ*) [–], which is mainly dominated by the pressure loss of the membrane, can be obtained through Eq. (6) in Additional file 1: Table S2. In Eq. (6), (*l*) in [*m*] is the nominal length of the module, given in Table 1.

The pressure losses as the function of the pump head over the generated permeate flow velocity for water, fermentation broth and sodium lactate for the feeds, water and fermentation broth are given in Fig. 1a–c. This type of representation allows for convenient analysis of the membrane performance (Marriott et al. 2001). Membranes with a low pressure loss have a flat curve and hence have a high permeate flow velocity at moderate pump head values. The performance will be evaluated by the pressure loss taken from Eq. (6) in Additional file 1: Table S2, which is directly proportional to the pump head and the permeate flow velocity through Eq. (1) in Additional file 1: Table S2 (Schock and Miquel 1987). The system curves are numbered in sequence as they appear in the Fig. 1a, b, and c from left to right. The number of each system curve can be found at the top of the diagram near the frame, right on the dotted line. Each system curve has its own pressure loss. Membrane points located on the same system curve share the same pressure loss at different pump head values (Karassik 2008). A maximum of six pump head levels were applied, 50–300 m, in 50 m steps, with 50 m being the first and 300 m the sixth level, or rather the sixfold pump head height. For the sake of clarity within the discussion of the membrane performance, the membrane points will be characterized by pump head level and the individual membrane pressure loss in percentage variance, compared with the individual membrane pressure loss at 50 m pump head. Both values will be given in round brackets divided by a slash, e.g., (1/60%).

The recovery for ions and impurities is calculated by Eq. (7) in Additional file 1: Table S2. In Eq. (7) (*C*_*F*_) in [ppm] is the concentration of the individual substance in the feed-tank and (*C*_*P*_) in [ppm] in the permeate stream.

### Upstream processing of sodium lactate

The hydrolysate was prepared from 75 kg of tapioca starch and 10.5 kg of yeast extract. The temperature was set to 80 °C, and the pH level was set to pH 6 by addition of 20% NaOH. Then, 75 ml of the enzyme BAN (*Bacillus amyloliquefaciens* α-amylase) from the Novozymes company (Bagsværd, Denmark) and 75 g of CaCl_2_ were added and stirred for 20 min, using a stirrer velocity of 137 rpm with a pitched 3-blade impeller. The hydrolysate was thermally sterilized and then filtered. The overall permeate volume was 240 l.

For the fermentation, LA bacteria *Bacillus coagulans*—St. A59 was added, and the temperature was set to 52 °C for 69 h. The volume at the beginning of the fermentation process was 708 l. During the fermentation process, 108 l of 20% NaOH solution was continuously added to maintain a pH of 6.0. After the fermentation process, the fermentation broth was sterilized. The sterilization was carried out at 119 °C for 45 min.

The inactivated fermentation broth was microfiltered using 2× CèRAM INSIDE from TAMI, Germany. The membrane material was ZrO_2_–TiO_2_ with a pore size of 0.2 µm and a total area of 6.65 m^2^ (Tami INSIDECéRAM. *Ceramic Tubular Membranes*). The batch was split into charges of 60 l each and frozen for further processing. The microfiltered fermentation broth was characterized by a total nitrogen concentration of 944 ppm, a total phosphate concentration of 99 ppm, a nitrite content of 0.5 ppm, a volume of 900 l, a conductivity of 30 mS cm^−1^, a pH of 6, and a sodium lactate concentration of 6000 ppm. The concentration profiles of the ions after the microfiltration are given in Table 2.

**Table 2:** Ion concentration of the microfiltered fermentation broth and the ion rejection of the membranes at 30 bar (NP30 at 25 bar)

Ions [–]	Concentration (ppm)	Rejection (%)					
		DK73	DL73	NP30	NF45	TW30	SW30
Mg^2+^	23	89	96	76	90	44	75
Ca^2+^	88	78	89	71	89	23	63
K^+^	357	16	11	66	49	41	69
Na^+^	18,220	18	3	65	49	43	69
NH_4_^+^	111	29	27	76	39	46	70
Cl^−^	112	−4	9	55	−25	25	39
SO_4_^2−^	153	71	64	65	88	26	61
PO_4_^3−^	11	74	55	56	100	−82	26

For a better understanding of the context, the inactivated and microfiltered fermentation broth will be referred to as “media” thorough the rest of the manuscript.

## Results and discussion

### Membrane pressure losses for water, media, and sodium lactate

To determine the optimal operating point of the membranes, the system curves of the membranes are plotted with the pump head as the function of the permeate flow velocity. The pressure losses were calculated according to the procedure described in “Market investigation, membrane selection and properties” section and compared by their percentage deviation starting with the first pump head level and then according to the rising pump head. This allows for a straightforward identification of the optimal operation point of each membrane by applied pump head and effective pressure loss.

**Fig. 2:**
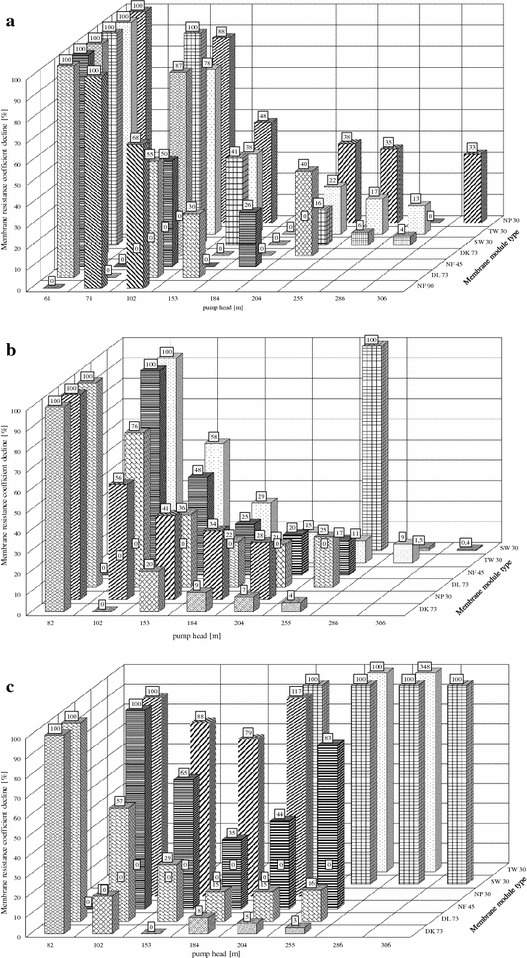
The percentage change in the membrane resistance coefficient of each membrane, obtained through the gradient of the system curves of the **a**, **b**, and **c** for every pump head. The values of the gradients are given in Additional file 1: Table S1. The figures allow for the identification of the optimum membrane resistance coefficient vs. pump head ratio for each individual membrane. For example, a 2.51-fold rise in the pump head, from 61 to 153 m, lead to a reduction of the membrane resistance coefficient to 30%, of the membrane DL73, whereas the membrane NP30 only reached 48%

Figure 1a shows the system curves of the membranes, with the pump head as the function of the applied permeate flow velocity using deionized water as the feed. The membranes DL73, DK73, and NF45 have decreasing pressure losses for increasing pump head, resulting in a flat curve. DL73 has the least pressure loss, with a doubling of the pump head decreasing the pressure loss by one-half (102 m/55%) and the tripling of the pump head decreasing the pressure loss (153 m/30%) to one-third; see Fig. 2a.

The membranes NP30, TW30, and SW30 only show a limited effect of the pump head on the pressure loss, resulting in steep slope in their system curve. The pressure loss of membrane NP30 remains at 88% at a pump head of 102 m. The pressure loss decreases to 48% at 153 m, but increasing the pump head to 204, 255, and 306 m had little effect on the pressure loss, with pressure loss measurements of 38, 35, and 33%, respectively.

Figure 1b shows the system curves of the membranes, as the function of the pump head over the applied permeate flow velocity, using fermentation broth as the feed. The impact of the pump head on the permeate volume flow rate is more noticeable when fermentation broth is used as the feed than when water is used as the feed. Hence, the system curves for almost all of the membranes are flat and the system curves are shifted towards higher head pressures. This means that no permeate flow was observed until the pressure valve was adjusted to 82 and 102 m for the membranes DK73, NP30, DL73, and TW30, SW30.

Despite the different basic pressure levels, all membranes show a significant effect on the pump head within the first two pump head levels. The pressure losses from 82 m to 102 m for NP30 and DL73 are reduced to 56% and 76%, respectively, see Fig. 2b. At higher pump head levels, all membranes share similar reductions in pressure losses; at 204 m, the pressure losses are 7, 28, 21, 20, and 15% for the membranes DK73, NP30, DL73, NF45, and TW30, respectively.

A further increase of the pump head leads did not lead to a significant reduction of the pressure loss. At a pump head level of 255 m, none of the membranes gained an additional pressure loss of more than 4%. It can therefore be concluded that the optimal operating pump head for the membranes DL73, TW30, and NF45 is the 184 m level, whereas the membranes NP30 and DK73 can be best operated at a pump head level of 153 m.

The best performance was shown by the membrane DK73, where a shift from 82 to 153 m in the pump head leads to a reduction to 20% in the pressure loss; see Fig. 2b.

The pressure loss originates from the structural change of the polymer thin layer membrane material, which is being compressed in a dense form by the applied feed pressure of the feed stream. This is expressed through the rise in the overall membrane resistance coefficient. The electrical power consumption of the pump is now increasingly being used to circulate the feed stream than over the membrane and back into the feed tank, rather than processing the feed stream though the membrane an increasing hereby the volume of the desired permeate stream.

### Lactate permeability

The lactate permeability is the most important feature of the membrane, as high lactate permeability allows for the purification of a greatest quantity of lactate for a given membrane area. Figure 1c shows the pump head as a function of the applied permeate flow velocity of sodium lactate using fermentation broth as the feed. As the LA is only a small part of the fermentation broth, the permeate flow velocity of the LA within the fermentation broth is also quite small, and therefore, the system curves are steep. In the case of SW30, the permeability of LA was limited. The pump head had no significant effect on the pressure loss. In three cases, increasing the pump head increased the pressure loss above the initial value. TW30 at 286 m had a 348% higher pressure loss than at 255 m. The pressure loss of NP30 shifted from 79% at 184 m to 117% at 204 m, and the pressure loss of NF45 shifted from 44% at 204 m to 83% at 255 m; see Fig. 2c. The optimum membrane and pump head for high LA permeate flow velocity would be the DL73 at 153 m, with a pressure loss of 29%, one-third of the initial pressure loss at 82 m and the highest overall LA flow, or the DK73 membrane with a pressure loss of 19% at 102 m. At higher pump head, no significant additional pressure loss is achieved.

The experiments demonstrated that the media, but not the sodium lactate permeate flow velocity are proportional to the pump head. Hence, the sodium lactate permeability had to be determined individually for each membrane. The sodium lactate permeability is equally important for the overall process performance of a membrane. Low lactate permeability leads to poor process performance. The strong influence of the pump head on the membranes’ lactate permeability makes investigation under real process conditions mandatory.

However, from the seven membranes investigated, two of the three membranes with the best water permeate flow velocity were also the membranes with best lactate permeate flow velocity. This result already indicates that the most important information on behalf of the membrane performance, the permeate flow velocity can be evaluated using deionized water as feed. The obtained order of the membranes performances according to permeate flow velocity was not significantly altered by the change of the feed material from deionized water to fermentation broth. Therefore, deionized water, using the presented method, can already be adequate feed material for the membrane evaluation if the desired process material is not available in sufficient quantities, due to limited storage capacities or a great amount of membranes to be investigated.

The presented tools for the evaluation of the membrane module performance via the measurement of the permeate flow velocity in dependency of the applied feed pressure (pump head) followed by the graphical method for determination of the gradient of the system curves and the mathematical description to obtain the membrane resistance coefficient form this gradient proved to be a valuable tool for the bio-process engineer to determine the best membrane module within a low number of experiments.

### Ion rejection

The quality of the generated permeate was investigated. The transport of charged molecules through the membrane produced a polarization of the membrane surface. Charged ions could diffuse through negatively charged membranes, but they were repelled by the positively charged membranes. Each membrane had a different rejection characteristic, which was expressed through the ion rejection and the organic molecule rejection.

Table 2 shows the ion rejection of the membranes. The membranes showed very different ion rejection characteristics. They demonstrated the Donnan effect, which described the electric potential equilibrium across the cross section of the membrane. Bivalent ions were more strongly retained than monovalent ions. Of the bivalent ions, the cations (magnesium, calcium) were more strongly retained than the anions (sulfate, phosphate). The anions were pushed through the membrane to maintain potential equilibrium (Donnan 1995). This resulted in a negative rejection in the case of chloride (DK73, NF45) and phosphate (TW30). The Donnan effect was partly compensated by the processing of the negatively charged lactate through the membrane, which contributed to the potential equilibrium. The low rejection of sodium provided evidence to support this thesis. The lowest sodium and potassium rejection was produced by DL73 and DK73, the membranes with the highest sodium lactate permeability. Both membranes had similar rejection characteristics. Three membranes (DL73, DK73, and NF45) produced good rejections for magnesium and calcium. The best average ion rejection was achieved by the NP30, where all ions were rejected by a minimum of 55% or higher. The three reverse osmosis membranes (NF45, TW30, and SW30) did not produce equally desirable ion rejection as the membranes that were designed for acid processing (DL73, DK73, NP30).

### Organic solute rejection

Additional file 1: Figure S2 (see supporting information) shows the electropherograms generated by the CE system of the media permeate for the membranes and the feed (Laube et al. 2016). The electropherograms portray the rejection characteristic of the organic molecule fractions in the permeate. The impurity fractions were made up of unknown organic molecules, so their concentration cannot be determined; however, their peak areas were set into an equivalent percentage of the lactate peak. Based on this relationship, the peak area purity was calculated. The sum of all peak areas equals 100%. Furthermore, the peak fractions are proportional to the polarity of the lactate. Impurity fractions close to the lactate peak did have similar polarity. The lactate peak did migrate at seven min, while the organic impurities migrated between three and six min. Additional file 1: Figure S2 illustrates that the feed impurity fractions were made of two minor single peaks, at 3.5 and 4.1 min, and three medium double peaks at 3.9, 4.3, and 5.4 min. The feed purity, calculated according to the total mesh area, was 81%. By comparing the media permeate peaks of the membranes with the feed peaks, the following results became apparent. Membranes with a high purity either had a lactate peak much greater than the impurity peaks, such as DL73 (70.6%), or rejected an impurity double peak at approximately 5 min, such as DK73 (79.5%) and NF45 (78.8%). Membranes with a lower permeate purity did not reject this specific fraction, e.g., NP30 (72.2%), SW30 (71.6%) at 30 bar and TW30 (64.7%) at 30 bar.

In spite of (or actually due to) the fact that the applied mathematical description can be found in the common literature on pumps systems and the newly presented graphical method can be applied to all membrane module-based filtration systems, we do believe that other engineers will apply our convenient method to assess their portfolio of possible membrane modules according to the membrane module most suitable for their filtration task.

## Conclusions

In this study, the optimal pump head of seven commercially available nanofiltration membranes (NF90, DL73, NF45, DK73, SW30, TW30, and NP30) for the purification of sodium lactate from fermentation broth was investigated. The experiments on fermentation broth permeability and sodium lactate permeability showed that DL73 and NP30 produced good results. When fermentation broth was used as the feed, DL73 and NP30 achieved pressure losses of 22 and 28% at a pump head of 184 and 204 m, respectively. However, the DK73 had the overall best performance according to the measured fermentation broth permeability and LA permeability, with pressure losses of 20 and 8% at a pump head of 153 and 184 m, respectively.

## Additional file

**Additional file 1: Figure S1.** Schematic set up of the cross-flow filtration plant with (PI) for pressure indicator, (FI) for flow through indicator, and (TI) for temperature indicator. **Figure S2**. Electropherograms generated by the CE system of the feed and the membranes media permeate at 30 bar (NP30 at 25 bar). **Table S1.** Membrane resistance coefficients for all system curves of water, media and LA. **Table S2.** Equations.
