# Supplementary material for: Investigation of spiral-wound membrane modules for the cross-flow nanofiltration of fermentation broth obtained from a pilot plant fermentation reactor for the continuous production of lactic acid
Source: Bioresour Bioprocess. 2017 Jan 4;4(1):4. doi: 10.1186/s40643-016-0133-5 (PMC5236076; doi:10.1186/s40643-016-0133-5)
Supplement: Supplementary file 1 — Additional file 1: Figure S1. Schematic set up of the cross-flow filtration plant with (PI) for pressure indicator, (FI) for flow through indicator, and (TI) for temperature indicator. Figure S2. Electropherograms generated by the CE system of the feed and the membranes media permeate at 30 bar (NP30 at 25 bar). Table S1. Membrane resistance coefficients for all system curves of water, media and LA. Table S2. Equations. [file 40643_2016_133_MOESM1_ESM.docx]

## Supporting Information

**Selection of spiral wounded pilot plant sized cross-flow filtration membranes for the purification of fermentative produced sodium lactate**

Hendrik Laube^1*^, Roland Schneider^1)^, Joachim Venus^1)^

^1^Department of Bioengineering, Leibniz-Institute for Agricultural Engineering Potsdam-Bornim e.V., Potsdam, Brandenburg, Germany

**Correspondence:** Department of Bioengineering, Leibniz-Institute for Agricultural Engineering Potsdam-Bornim e.V., Max-Eyth-Allee 100, D-14469 Potsdam, Germany

Tel/Fax: +49-(0331)-5699-121, E-mail: [hlaube@atb-potsdam.de](mailto:hlaube@atb-potsdam.de)

## Descriptive legend

**Supplementary Table S1** Membrane resistance coefficients for all system curves of water, media and LA

**Supplementary Table S2** Equations

**Supplementary Figure S1** Schematic set up of the cross-flow filtration plant with (PI) for pressure indicator, (FI) for flow through indicator, and (TI) for temperature indicator

**Supplementary Figure S2** Electropherograms generated by the CE system of the feed and the membranes media permeate at 30 bar (NP30 at 25 bar)

| Supplementary Table S1 | | | |
| --- | --- | --- | --- |
| No | System Curve Coefficients | | |
| [-] | Water [10^14^] | Media [10^15^] | LA [10^17^] |
| 1 | 4.31 | 500.65 | 3530 |
| 2 | 1.77 | 8.48 | 28.3 |
| 3 | 1.27 | 4.94 | 21.2 |
| 4 | 0.989 | 3.39 | 8.12 |
| 5 | 0.706 | 2.47 | 6.71 |
| 6 | 0.480 | 1.62 | 5.30 |
| 7 | 0.283 | 1.27 | 4.03 |
| 8 | 0.247 | 0.918 | 3.60 |
| 9 | 0.212 | 0.848 | 2.83 |
| 10 | 0.170 | 0.777 | 1.98 |
| 11 | 0.134 | 0.671 | 1.70 |
| 12 | 0.106 | 0.586 | 1.48 |
| 13 | 0.0989 | 0.516 | 1.34 |
| 14 | 0.0918 | 0.318 | 1.13 |
| 15 | 0.0671 | 0.290 | 1.08 |
| 16 | 0.0424 | 0.240 | 0.657 |
| 17 | 0.0346 | 0.212 | 0.565 |
| 18 | 0.0233 | 0.173 | 0.311 |
| 19 | 0.0127 | 0.152 | 0.297 |
| 20 | - | 0.145 | - |
| 21 | - | 0.113 | - |
| 22 | - | 0.0784 | - |
| 23 | - | 0.0706 | - |
| 24 | - | 0.0671 | - |

| Supplementary Table S2 | |
| --- | --- |
| $H_{Jt}=\frac{\vartheta^{2}}{2\cdot g}\cdot\left( \frac{\lambda\cdot L}{d_{i}}+\sum\xi\right)$ | (1) |
| $f\left( x \right)=a\cdot x^{2}$ | (2) |
| $\lambda=\frac{0.309}{\left( lg\frac{{Re}_{d}}{7} \right)^{2}}$ | (3) |
| ${Re}_{d}=\frac{\vartheta_{i}\cdot d_{i}}{v}=\frac{\vartheta_{i}\cdot d_{i}\cdot\rho}{\eta}=\frac{\dot{m}\cdot d_{i}}{A\cdot\eta}$ | (4) |
| $a=\frac{1}{2\cdot g}\cdot\left( \frac{\lambda\cdot L}{d_{i}}+\sum\xi\right)$ | (5) |
| $\sum\xi=2\cdot g\cdot a-\frac{\lambda\cdot L}{d_{i}}$ | (6) |
| $R\left( \% \right)=\left( 1-\frac{C_{F}}{C_{P}} \right)\cdot100\%$ | (7) |

|  |
| --- |
| Supplementary Figure S1 |

|  |
| --- |
| Supplementary Figure S2 |
